# Comprehensive Analysis of Metabolome and Transcriptome Reveals Physiological Processes Related to Larval Development of Barnacles (*Megabalanus volcano*)

**DOI:** 10.3390/ani16030413

**Published:** 2026-01-28

**Authors:** Zewen Zheng, Duo Chen, Ziquan Zhou, Siwen Peng, Xuehui Li, Zhenyi Zhuang, Haiyan Yao, Xiaozhen Rao, Ting Xue, Gang Lin

**Affiliations:** Fujian Key Laboratory of Special Marine Bioresource Sustainable Utilization, College of Life Sciences, Fujian Normal University, Fuzhou 350117, China; zhengzewen2019@163.com (Z.Z.); chenduo@fjnu.edu.cn (D.C.); weatherzzq@163.com (Z.Z.); 13509585056@163.com (S.P.); 15159013962@163.com (X.L.); 15859581876@163.com (Z.Z.); daphneyanng@163.com (H.Y.); xzrao@fjnu.edu.cn (X.R.); xueting@fjnu.edu.cn (T.X.)

**Keywords:** *Megabalanus volcano*, development, metabolome, transcriptome

## Abstract

Background: Barnacles are important marine fouling organisms, and their complex life cycle involves key metamorphic nodes from nauplius to cyprid larvae and then to sessile adults. However, the molecular mechanisms underlying their larval development remain poorly understood. Metabolomics and transcriptomics are powerful tools for exploring biological development pathways and regulatory networks. Methods: We employed non-targeted metabolomics and transcriptomics to analyze three key developmental stages of embryonic stage, nauplius stage, and cyprid stage. Differential metabolites were screened using fold change (FC), *p*-value, and variable importance in projection (VIP) values, while DEGs were identified with adjusted *p*-value and |log_2_(fold change)| criteria. WGCNA was used to construct gene co-expression networks, and qRT-PCR validated RNA-seq results. Results: A total of 3683 metabolites were identified, with the bile secretion pathway serving as a core regulatory pathway throughout early development. Transcriptomic analysis identified 7234 DEGs, which were clustered into four modules corresponding to different developmental stages. Key pathways such as chitin metabolism, and linoleic acid metabolism were significantly enriched, and qRT-PCR confirmed the reliability of RNA-seq data. Conclusions: This study reveals the metabolic and molecular regulatory mechanisms underlying the early development of *M. volcano*, highlighting stage-specific metabolic characteristics and core gene modules. The findings provide a theoretical basis for understanding barnacle developmental adaptation strategies and offer potential targets for the development of novel antifouling agents.

## Simple Summary

Scientists need to understand how barnacles develop because these small marine organisms are a type of harmful fouling organism; they attach to ships, marine infrastructure, and aquaculture facilities, causing huge economic losses. Barnacles have a complex life cycle, and the transformation from their nauplius larval stage to cyprid larval stage is key, but the biological processes behind this are not well known. This study used two scientific methods—metabolome and transcriptome analysis—to look at three key developmental stages of the barnacle *Megabalanus volcano*: embryo, nauplius larva, and cyprid larva. The research identified 3683 distinct metabolites and 7234 genes with altered activity across different stages. The bile secretion pathway was central to all early development stages, helping with lipid digestion and mineral absorption for the barnacles’ shape changes. The study also identified gene sets associated with each stage; for instance, genes related to the Wnt signaling pathway are active in cyprids, while chitin metabolism genes play a key role in nauplius. These findings help us understand how barnacles grow and adapt to their environment.

## 1. Introduction

In the vast marine ecosystem, barnacles, as unique marine organisms, are widely distributed across oceans worldwide [1]. Their presence can be found from the cold polar seas to the warm tropical waters found in seas ranging from frigid polar oceans to warm tropical waters [2,3]. Though small in size, these tiny arthropods hold a special position in the marine ecosystem [4,5]. On one hand, they serve as a critical prey source for zooplankton and a core component of benthic communities, participating in the cycling of the marine food chain. On the other hand, their specialized attachment behavior directly influences the biogeochemical cycling processes of marine substrates [6]. Barnacles have a distinctive survival strategy they are like “mysterious tenants” of the ocean, often attaching themselves to the surfaces of various objects. Marine organisms, ships, and reefs are all their preferred habitats [7,8]. Precisely because of this trait, they are regarded as highly harmful marine fouling organisms [9,10,11]. Their presence exerts significant impacts on marine infrastructure projects, aquaculture, and ship transportation, and causes considerable economic losses [12,13]. It is reported that the U.S. Navy spends hundreds of millions of dollars annually on the management of marine fouling organisms [14,15,16].

Barnacles possess a complex life cycle, encompassing a planktonic larval stage and a sessile adult stage [17,18]. The planktonic stage includes the nauplius phase and the cyprid phase; the sessile stage mainly refers to the cyprid larvae searching for suitable attachment sites via their antennae, then developing into adults and secreting adhesive substances to achieve permanent attachment [10,19]. Throughout the entire life cycle, two key metamorphic events occur: the transformation from nauplius to cyprid larvae, and the sessile metamorphosis of cyprid larvae into adults [20,21]. Barnacles belong to the group of organisms with indirect development; their larvae exhibit striking differences from adults in terms of morphological structure, locomotion mode, feeding habits, and habitat preferences. Unlike species with direct development, barnacle larvae must undergo 6–8 molts and multiple rounds of morphological remodeling to complete the transition from a “planktonic” to a “sessile” lifestyle [17].

Their core life cycle can be clearly outlined as follows: Egg–Nauplius Larva–Cyprid Larva–Adult [22]. The egg masses are pale yellow in color and rely on water flow exchange within the maternal hemocoel to obtain oxygen and nutrients. Upon hatching, nauplius larvae emerge, detach from the mother, and enter the planktonic environment. This stage is characterized by a simple morphology typical of planktonic organisms; through successive molts, the larvae increase in size and their body structures become more complex [22]. Following the final molt, nauplius larvae transition into the cyprid larval stage—a critical transitional phase marking the shift from planktonic to sessile life. Cyprid larvae differ dramatically from nauplius larvae in morphology, signifying the official onset of metamorphic development [23,24]. Moreover, the biomechanical properties of their antennules are directly linked to their attachment and settlement behaviors [25].

Despite notable progress made in current barnacle research, including morphological observations, ecological adaptability analyses, and preliminary explorations of attachment mechanisms, the molecular regulatory networks governing their larval development remain poorly understood [17,19,22,26,27]. Moreover, existing research on *M. volcano* has only analyzed transcriptomic differences between cyprid larvae and adults, failing to cover the embryonic and nauplius larval stages [28]. For other barnacle species such as *Pollicipes pollicipes*, research lacks metabolomic data support [17]. These limitations have resulted in critical knowledge gaps in the study of the “metabolite-gene” coordinated regulatory networks underlying early barnacle development. Furthermore, most existing studies have focused on the model species *Amphibalanus amphitrite*, while research data on other dominant fouling species of significant ecological importance are extremely scarce. This gap limits our comprehensive understanding of the universality and specificity of developmental mechanisms across barnacle taxa.

In recent years, the combined application of metabolomics and transcriptomics has emerged as a pivotal technical approach for deciphering the development-related pathways and regulatory networks of arthropods. For instance, transcriptomic studies have uncovered the developmental and physiological mechanisms of the two-spotted spider mite (*Tetranychus urticae*) [29]; integrated transcriptomic and metabolomic analyses have elucidated the molecular mechanisms underlying “annual precocity” in the Chinese mitten crab (*Eriocheir sinensis*) [30]; and multi-omics integration techniques have resolved the regulatory differences affecting growth performance in the Pacific white shrimp (*Litopenaeus vannamei*) [31]. However, in the field of barnacle research, few studies have integrated these two technologies to investigate the physiological processes associated with development from the embryonic stage to the cyprid larval stage.

This study selects *M. volcano* as the research subject. As a dominant fouling species in China’s coastal waters, it boasts a broad distribution range and strong adaptive capacity, exerting significant adverse effects on local marine aquaculture and shipping industries, thus rendering it of substantial research value. Meanwhile, as a species distinct from the extensively studied Balanus *A. amphitrite*, investigating its developmental mechanisms will fill the research gap regarding non-model barnacle species and provide fundamental data for comparative studies on the evolutionary conservation and divergence of barnacle developmental regulatory networks. Employing metabolomic and transcriptomic techniques, this research examines the developmental process of barnacles from egg masses and nauplius larvae to cyprid larvae. By analyzing differential metabolites and differentially expressed genes (DEGs) across different developmental stages, this study screens and identifies pathways and genes associated with specific developmental phases. The findings of this research will provide a crucial foundation for further unraveling the biological mechanisms that underpin barnacle development.

## 2. Materials and Methods

### 2.1. Acquisition of Developmental Samples of M. volcano

**Figure 1:**
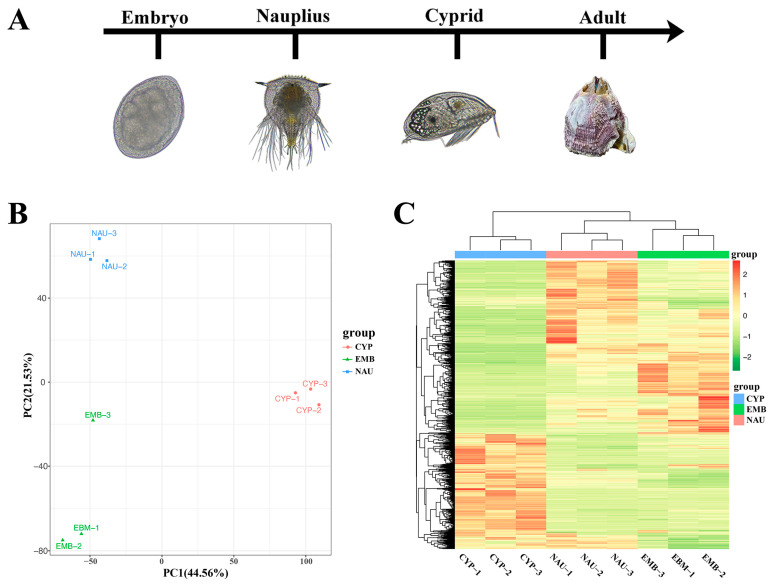
(**A**) Diagram representing the experimental design. Three developmental stages (three replicates per time point) were selected: embryonic stage; nauplius stage; and cypris stage. For the images of the studied developmental stages, the scale bar in each image represents 100 μm. (**B**) Principal component analysis of metabolites in samples from different developmental stages. (**C**) Cluster analysis of metabolites in samples from different developmental stages.

In this study, *M. volcano* was used as the experimental subject to systematically analyze the dynamic changes in metabolome and transcriptome across three key developmental stages, namely the embryonic stage (EMB), nauplius stage (NAU), and cyprid stage (CYP) (Figure 1A). The *M. volcano* specimens used in the experiment were collected from offshore aquaculture rafts in Pingtan County, Fujian Province, China (geographic coordinates: 26.0173° N, 119.6653° E). After collection, 150 adult barnacles from different parent stocks were temporarily reared together in a 150 L incubator in the laboratory. During the temporary rearing period, water was changed regularly, and *Chaetoceros muelleri* was fed as bait after each water change.

The temporarily reared barnacles were subjected to 6 h of air exposure, followed by flowing water stimulation to induce egg mass release. After collecting the released pale-yellow egg masses, they were immediately observed under a microscope to select those with intact morphology and at the gastrula stage. The qualified egg masses were rinsed three times with sterile seawater to remove surface impurities, then randomly and equally divided into three portions and transferred into sterile centrifuge tubes, with approximately 0.5 g of wet weight per tube, which served as three biological replicates for the embryonic stage every day. Leveraging the phototaxis of nauplius, a spotlight was used to concentrate the larvae. The nauplius released by parent barnacles were then siphoned into a new 50 L culture tank for separate rearing. No bait was provided during this developmental stage, and the culture conditions were maintained as follows: water temperature of 25 °C, salinity of 30‰, and a 24-h dark photoperiod. The larvae were continuously cultured until they developed to the Nauplius Ⅲ stage. Nauplius Ⅲ individuals were collected using a 100-mesh plankton net. Subsequently, the net was rinsed three times with sterile seawater to elute the larvae into a 500 mL sterile beaker. After counting, the larval density was adjusted to 3 ind/mL. Thereafter, the larvae were re-concentrated using the 100-mesh plankton net again and thoroughly rinsed five times with sterile seawater to remove residual impurities. Finally, the larvae were aliquoted into 1 mL centrifuge tubes, with approximately 1500 larvae per tube, and three parallel biological replicates were set up. Notably, the Nauplius Ⅲ stage represents a critical phase in the developmental process of nauplius; during this stage, the larvae complete the shift from endogenous nutrition to the preparation phase for exogenous nutrition [22]. Since no exogenous feeding was performed during this period, larval development was arrested at the Nauplius Ⅲ stage, and this developmental arrest ensured excellent homogeneity of the samples.

The remaining nauplius were transferred to a 50 L culture tank for further rearing under the following conditions: water temperature of 25 °C, salinity of 30‰, a 24-h dark photoperiod, and larval density of 1 ind/mL. *C. muelleri* was fed daily at an algal density of 8 × 10^4^ cells/mL. During the rearing period, one-third of the culture water was replaced daily, and residual feed and contaminants at the bottom of the tank were promptly removed. The larvae were continuously cultured until they completed the final molt and metamorphosed into cyprids. Cyprids were concentrated using a spotlight and subsequently collected with a 120-mesh plankton net. After rinsing four times with sterile seawater, the collected samples were filtered through an 80-mesh plankton net. The nauplius retained on the 80-mesh net were transferred back to the culture tank for further rearing, while the cyprids filtered through the net were moved into a 500 mL sterile beaker. After counting, the cyprid density was adjusted to 3 ind/mL, followed by a second concentration step using a 120-mesh plankton net. The cyprids were then thoroughly rinsed five times with sterile seawater to eliminate residual impurities, randomly aliquoted into 3 centrifuge tubes, with approximately 1500 larvae per tube, and designated as three biological replicates for the cyprid stage.

All samples in the centrifuge tubes were homogenized, immediately frozen in liquid nitrogen for quick-freezing, and then stored in a −80 °C refrigerator for subsequent analysis.

### 2.2. Metabolomics Analysis of Developmental Samples of M. volcano

Non-targeted metabolomics was employed to analyze metabolites across the three early developmental stages of barnacles. For the experiment, a Waters Acquity I-Class PLUS ultra-high performance liquid chromatography (UHPLC) system (Waters Corporation, Milford, MA, USA) equipped with a Waters Acquity UPLC HSS T3 column (100 × 2.1 mm, 1.8 μm; Waters Corporation, MA, USA) was used. This system was coupled with a Waters Xevo G2-XS QToF high-resolution mass spectrometer (Waters Corporation, MA, USA) and an ESI ion source for ultra-high performance liquid chromatography-tandem mass spectrometry (UHPLC-MS/MS) detection.

Chromatographic conditions: The mobile phase A was set as an aqueous solution containing 0.1% formic acid, and mobile phase B was acetonitrile solution containing 0.1% formic acid, under both positive and negative ion modes. The injection volume was 1 μL. The gradient elution procedure was performed at a constant flow rate of 400μL/min: the ratio of 98% A–2% B was maintained for 0–0.25 min, linearly switched to 2% A–98% B from 10 to 13 min, and restored to the initial ratio (98% A–2% B) during 13.1–15 min. Mass spectrometric conditions: Mass spectrometry data acquisition was controlled by MassLynx V4.2 (Waters) software. The MSe mode was adopted to synchronously collect primary and secondary mass spectrometry data, with the low collision energy set at 2 V, high collision energy ranging from 10 to 40 V, and a scanning frequency of 0.2 s per spectrum. The parameters of the ESI ion source were as follows: capillary voltage at 2500 V (positive ion mode)/−2000 V (negative ion mode), cone voltage at 30 V, ion source temperature at 100 °C, desolvation gas temperature at 500 °C, purge gas flow rate at 50 L/h, desolvation gas flow rate at 800 L/h, and the mass-to-charge ratio (*m*/*z*) acquisition range was 50–1200.

Raw data were acquired using MassLynx V4.2 software, followed by data processing steps such as peak extraction and peak alignment via Progenesis QI V2.0 software. Metabolite identification was based on accurate mass-to-charge ratio (mass error < 50 ppm) and secondary mass spectra, achieved by matching against public databases including the Human Metabolome Database HMDB [32], MassBank [33], LipidMaps [34], mzCloud (https://www.mzcloud.org), and the Kyoto Encyclopedia of Genes and Genomes (KEGG, http://www.genome.jp/kegg/ (accessed on 25 January 2026)).

Principal component analysis (PCA) was used to evaluate the reproducibility of intra-group samples and quality control (QC) samples. To verify the statistical significance of differences in metabolic characteristics among groups, permutational analysis of variance (PERANOVA) was conducted on the whole metabolomics dataset prior to PCA dimensionality reduction, and PCA dimensionality reduction was implemented based on the preprocessed metabolomics data. Three quantitative methods were combined for cross-validation of outliers within each group, including the Mahalanobis distance test, where the Mahalanobis distance from each sample to the intra-group mean in the PC1-PC2 dimension was calculated, with the critical value of the chi-square distribution (degrees of freedom = 2, α = 0.05, critical value = 5.99) used as the threshold and samples with a distance exceeding this threshold identified as outliers; the Euclidean distance ratio test where the average Euclidean distance between an individual sample and other samples in the same group was calculated before computing its ratio relative to the total intra-group average distance and samples with a ratio >2.5 defined as outliers; and Hotelling’s T^2^ test, where the Mahalanobis distance was converted into an F statistic and samples with *p* < 0.05 determined as outliers. Additionally, a heat map of hierarchical clustering was constructed on Euclidean distance in the R package pheatmap (v1.0.2). The identified compounds were categorized and their pathway information was retrieved from the KEGG, HMDB, and LipidMaps databases. Fold changes (FC) were calculated and compared based on group information, while the Student’s *t*-test was applied to determine the statistical significance (*p*-value) of differences for each compound. The R package ropls (v1.6.2) was used to construct the orthogonal partial least squares-discriminant analysis (OPLS-DA) model, and 200 permutation tests were performed to verify the model’s reliability. The variable importance in projection (VIP) values of the model were calculated via multiple cross-validation. Differential metabolites were screened by combining three parameters from the OPLS-DA model: fold change, *p*-value, and VIP value. The screening criteria were set as |log_2_ FC| > 1, *p*-value < 0.05, and VIP > 1. Finally, the hypergeometric distribution test was used to calculate the significance of KEGG pathway enrichment for the differential metabolites.

### 2.3. Transcriptomic Analysis of Developmental Samples of M. volcano

The samples used for transcriptomic analysis were identical to those for metabolomic analysis, covering three developmental stages. The study utilized the *M. volcano* genome (https://doi.org/10.57760/sciencedb.24738) as the reference, and its functional annotation was completed via eggNOG-mapper (v5.0). Total RNA was extracted using TRIzol reagent (Life Technologies, Carlsbad, CA, USA). RNA sequencing (RNA-seq) library construction followed the Illumina TruSeq protocol, and sequencing was performed on the Illumina HiSeq 2000 platform (Illumina, San Diego, CA, USA), generating 150 bp paired-end reads for each sample. After raw paired-end RNA-seq data were filtered by FASTP (v0.23.2) to obtain clean reads, STAR (v2.7.10b) was used for alignment to the reference genome. Raw counts were calculated using StringTie (v2.0), and low-expression genes were filtered out [35]. We subsequently performed differential expression analysis based on the negative binomial distribution in the R package DEseq2 (v1.30.1), with raw counts as input. The transformed counts following variance-stabilizing transformation in DESeq2 were used to perform PCA with all expressed genes. we screened for DEGs using the criteria of adjusted *p*-value ≤ 0.05 and |log_2_(fold change)| ≥ 1.

### 2.4. Construction of Transcriptome Co-Expression Network

To identify key active genes in larval development, we screened DEGs using the criteria of adjusted *p* ≤ 0.01 and |log_2_(fold change)| ≥ 2. The construction of co-expression network modules was performed using the WGCNA (Weighted Gene Co-Expression Network Analysis) package (v1.72-1) in R [36]. First, all genes were imported into the WGCNA package. A total of 4183 genes were screened by the criteria of coefficient of variation (CV) > 0.5 and CV < standard deviation (sd). Finally, co-expression modules were obtained with the following parameter settings: power value of 4, minimum module size of 30, and merge cut height of 0.25. After calculating the module eigengene of each module, a correlation analysis between modules and traits was performed, where the traits corresponded to the different larval developmental stages, thereby clarifying the association patterns between larval developmental stages and gene modules.

### 2.5. Quantitative Real-Time PCR Analysis of Gene Expression

To validate the RNA-seq data, total RNA from each sample was reverse-transcribed into cDNA using HiScript Ⅲ Q RT SuperMix for qPCR (+gDNA wiper) (Vazyme, Nanjin, China). β-actin was used as the reference gene, and primers for each target gene were designed using Primer5 software (Table S1). RT-PCR reactions were performed on a T100 Thermal Cycler (BIO-RAD, Hercules, CA, USA) with the following conditions: 30 s at 95 °C, 30 s at 55 °C, and 1 min at 72 °C, for a total of 35 cycles. The relative expression levels were calculated using the 2^−ΔΔCt^ method [37].

## 3. Results

### 3.1. Dynamic Changes of Metabolites During Larval Development of M. volcano

To elucidate the metabolic regulation patterns during larval development of *M. volcano*, non-targeted metabolomics analysis was performed across the three developmental stages. Results showed that a total of 3683 annotated metabolites were identified in at least one tissue sample, including 2079 in positive ion mode and 1604 in negative ion mode (Table S2). Principal component analysis (PCA) was used to analyze metabolite differences among developmental stages. The PCA plot indicated that the first two principal components (PCs) explained 57.86% of the total variance, and all samples exhibited clear stratification according to their developmental stages (Figure 1B). The result of PERMANOVA showed that F = 2.49 and *p* = 0.0081, which further verified the significance of inter-group differences and indicated that there were statistical differences in the overall metabolomic characteristics among the three groups of samples. Outlier detection was performed on the metabolomic data of the three groups of samples; the results demonstrated that the Mahalanobis distances of all samples were 1.33, the Euclidean distance ratios ranged from 0.50 to 0.92, all of which fell within the normal ranges, and the *p*-value of Hotelling’s T^2^ test was 0.577 (>0.05). All samples were ultimately determined as “Normal” in terms of outlier status, indicating good intra-group sample reproducibility and the absence of outliers (Table S3). Hierarchical clustering analysis further revealed that the three developmental stages could be divided into three independent clusters based on differences in metabolite composition, suggesting stage-specific accumulation of metabolites during barnacle larval development (Figure 1C). Additionally, both PCA and hierarchical clustering analysis showed that the three biological replicates of each group were closely clustered, indicating high reproducibility and reliability of the experimental data. Statistical classification of the identified metabolites revealed the following main categories: 485 carboxylic acids and derivatives, 324 fatty acyls, 312 prenol lipids, 240 steroids and steroid derivatives, 234 organooxygen compounds, 111 glycerophospholipids, 104 benzene and substituted derivatives, 53 organonitrogen compounds, 40 indoles and derivatives, 34 macrolides and analogues, and 157 other compounds not classified into the above 10 major categories.

Orthogonal partial least squares-discriminant analysis (OPLS-DA) scores further confirmed significant differences between samples from different developmental stages. Results showed that the Q^2^ values of all pairwise comparisons were higher than 0.89, indicating the model had good predictive ability (Figure S1). Differential metabolites were screened based on variable importance in projection (VIP) ≥ 1 and |log_2_(fold change)| ≥ 1. After K-means clustering analysis of these differential metabolites, three co-expression clusters were formed (Figure S2). Among them, Cluster 1 (571 metabolites) showed a gradual increase with developmental time, Cluster 2 (405 metabolites) first increased and then decreased, and Cluster 3 (415 metabolites) showed a gradual decrease.

To explore the functional significance of these differential metabolites, KEGG enrichment analysis was performed for each metabolite cluster. Metabolites in Cluster 1 were significantly enriched in basic metabolism related to lipid synthesis and digestion, such as bile secretion, primary bile acid biosynthesis, and fatty acid metabolism (Figure S3A). Metabolites in Cluster 2 were significantly enriched in metabolic pathways including sulfur metabolism, sphingolipid metabolism, apoptosis, and cutin biosynthesis (Figure S3B). Metabolites in Cluster 3 showed enrichment characteristics in multiple pathways, including mineral absorption, lysine degradation, protein digestion and absorption, mannose-type O-glycan biosynthesis, and ABC transporters (Figure S3C).

**Figure 2:**
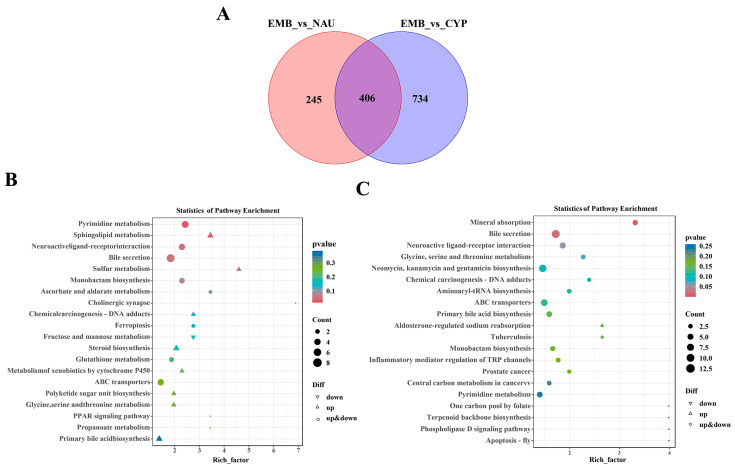
(**A**) Venn diagram of differential metabolites among different developmental stages. (**B**) KEGG enrichment term analysis of differential metabolites between egg and nauplius (top 20). (**C**) KEGG enrichment term analysis of differential metabolites between egg and cyprid (top 20).

Taking the EMB as the control group, comparative analyses between EMB and NAU, as well as between EMB and CYP, identified 651 and 1140 differential metabolites, respectively (Figure 2A). KEGG pathway enrichment analysis showed that in the EMB vs. NAU comparison, five pathways were significantly differentially enriched: pyrimidine metabolism, sphingolipid metabolism, neuroactive ligand–receptor interaction, bile secretion, and sulfur metabolism (Figure 2B). However, in the EMB vs. CYP comparison, mineral absorption and bile secretion emerged as the most prominently enriched pathways (Figure 2C). The above results indicate that the bile secretion metabolic pathway plays a core role in the early larval development of barnacles, and its activity gradually increases as larval development progresses.

### 3.2. Transcriptomic Dynamic Changes During Larval Development of M. volcano

**Figure 3:**
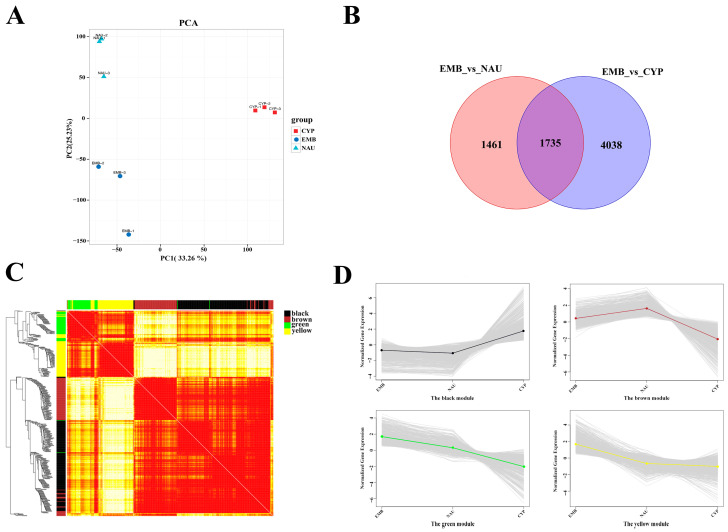
(**A**) Principal component analysis of gene expression in samples from different developmental stages. (**B**) Venn diagram of differential genes among different developmental stages. (**C**) Visualization of gene expression network using a heatmap plot (red tones indicate a positive correlation, and yellow tones a negative correlation). (**D**) Four clusters of the gene expression pattern.

A total of 62.62 Gb of clean sequencing data was obtained from nine samples, and details regarding the RNA sequencing data of each sample are provided in Table S4. After filtering the low-quality reads in the library, each sample yielded an average of 6.96 Gb of clean reads, with the percentage of Q30 bases exceeding 92.15%. These clean sequencing data were aligned to the *M. volcano* genome. PCA showed a high degree of consistency in gene expression patterns among samples from different biological replicates (Figure 3A). By comparing data from different developmental stages, we screened for DEGs using the criteria of adjusted *p*-value ≤ 0.05 and |log_2_(fold change)| ≥ 1. A total of 7234 DEGs were identified, and their number showed an increasing trend with the progression of larval development (Figure 3B).

To identify the key active genes in larval development, we filtered DEGs using adjusted thresholds of *p* ≤ 0.01 and absolute |log_2_(fold change)| ≥ 2. Through WGCNA, a total of 4183 DEGs were identified and ultimately classified into four distinct gene modules (Figure 3C). Among these modules, the black module containing 1309 DEGs was slightly downregulated in NAU but significantly upregulated in CYP, while the brown module with 654 DEGs was upregulated in the nauplius stage and downregulated in the cyprid stage relative to the nauplius stage. The 498 genes in the green module and 799 genes in the yellow module were notably downregulated in the nauplius stage and further downregulated in the cyprid stage (Figure 3D). “Module-trait correlation analysis” serves as a critical step in the WGCNA workflow, bridging module construction and biological interpretation, and we utilized each developmental stage to analyze the correlation between modules and traits. The black and yellow modules exhibited significant correlations with CYP, indicating that the high expression of genes in these modules was primarily concentrated in the cyprid stage. The brown module showed a significant correlation with NAU, suggesting its core function is closely associated with nauplius stage development. In contrast, the green and yellow modules displayed significant correlations with EMB. A detailed heatmap illustrating the correlations between gene modules and developmental phenotypes is presented in Figure S4.

**Figure 4:**
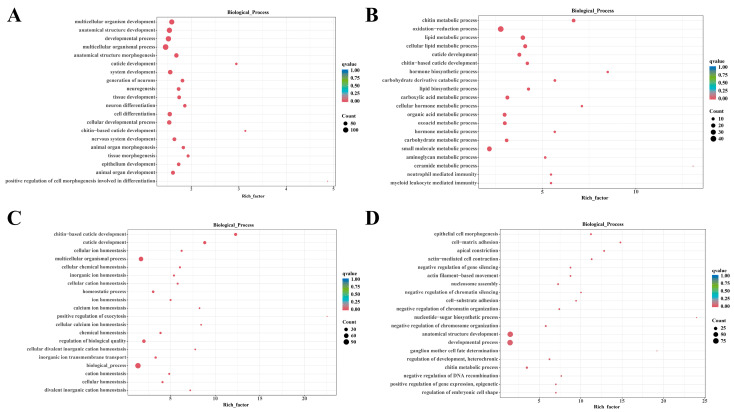
Top 20 GO terms of biological processes in the WGCNA modules. Enriched GO of the black module (**A**), the brown module (**B**), the green module (**C**), and the yellow module (**D**).

To further explore the biological functions of each module, we performed Gene Ontology (GO) and KEGG enrichment analyses, and screened for significantly enriched terms and pathways using *p* ≤ 0.05 as the threshold (Figures S5–S7). The top 20 GO terms in the “biological process” category were selected for visualization (Figure 4). Results showed that the DEGs in the black module were mainly involved in biological processes related to growth and development—such as multicellular organism development, anatomical structure development, and developmental processes—especially those associated with the nervous system, tissue/organ formation, and cell differentiation. KEGG analysis further confirmed that this module was also significantly enriched in pathways including the Wnt signaling pathway and Phototransduction-fly pathway. The DEGs in the brown module had scattered functional distributions, with significant enrichment in multiple metabolic processes: chitin metabolism, redox reactions, lipid metabolism, cuticle development, hormone synthesis and metabolism, carbohydrate metabolism, and small molecule metabolism. The DEGs in the green module were significantly enriched in biological processes related to cuticle development and ion/chemical homeostasis regulation, such as chitin-based cuticle development and cellular ion homeostasis. The DEGs in the yellow module were mainly significantly enriched in various biological processes, including epithelial cell morphogenesis, cell adhesion, actin-mediated cellular processes, chromatin and chromosome-related regulation, nucleotide-sugar biosynthesis, and embryonic cell morphology regulation.

### 3.3. Expression Patterns of Key Components

The metabolism and accumulation of bioactive and nutrient compounds in barnacle larvae are crucial for metamorphic development. Notably, KEGG enrichment analysis revealed that the DEGs in the brown module were significantly enriched in biosynthetic and metabolic processes of various substances, including amino acid and nucleotide sugar metabolism, thiamine metabolism, folate biosynthesis, lipid metabolism, and the pentose phosphate pathway. By analyzing the co-regulation network of these genes, the mechanism of their synergistic action in developmental regulation can be further clarified. To verify the analytical value of the co-regulation network in the regulation of early barnacle larval development, this study focused on analyzing the gene expression patterns of the following two key pathways.

Amino acid and nucleotide sugar metabolism provide key precursors for chitin synthesis. During the development of barnacle larvae, a total of 15 genes directly related to chitin metabolism were identified. Among them, chitinase genes showed the highest abundance with 11 gene copies. In addition, key synthetic genes such as UDP-N-acetylglucosamine/UDP-N-acetylgalactosamine diphosphorylase genes and chitin synthase genes were also clustered in this co-expression module (Figure S8), suggesting a coordinated expression regulatory relationship of these genes in the chitin metabolism process.

Furthermore, linoleic acid, as an essential fatty acid, plays an important role in cell membrane construction, signal molecule synthesis, and energy supply. A total of 12 genes involved in linoleic acid metabolism were identified in this study. Some of these genes overlapped in function with other lipid metabolism genes, including secretory phospholipase genes, cytochrome P450 family 2 subfamily J (CYP2J) genes, and cytochrome P450 family 3A (CYP3A) genes. These genes are distributed in an orderly manner in the linoleic acid metabolism pathway, collectively regulating the oxidative decomposition of linoleic acid and the synthesis of its derivatives (Figure S9).

### 3.4. Validation of RNA Sequencing Results by RT-PCR

**Figure 5:**
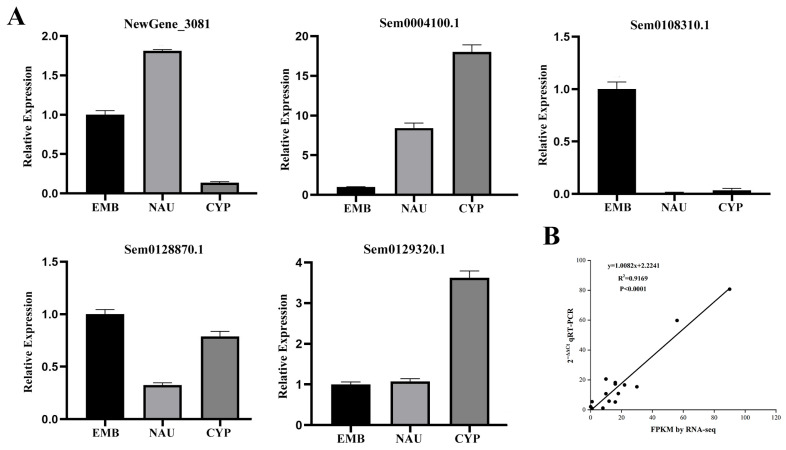
(**A**) Relative expression of five genes at different developmental stages detected by qRT-PCR. (**B**) Correlation analysis between RNA-Seq and qRT-PCR data.

To verify the accuracy of RNA sequencing data, we randomly selected five genes for quantitative real-time PCR (qRT-PCR) detection (Figure 5). Results showed that this technique could accurately reflect the expression levels of the relevant genes. The gene expression trends were consistent with the RNA-Seq analysis results. Correlation analysis between RNA-Seq and qRT-PCR data confirmed the reliability of the RNA-Seq data.

## 4. Discussion

This study systematically elucidated the early developmental mechanisms of *M. volcano* from the embryonic stage and nauplius stage to the cyprid stage using metabolomics and transcriptomics. It revealed the key regulatory pathways and material bases underlying the “egg–nauplius larva–cyprid larva” developmental process at the molecular and metabolic levels, providing a new perspective for understanding the adaptive strategies of metamorphic development in marine arthropods.

### 4.1. Dynamic Changes in the Metabolome Reflect Differences in Functional Demands Across Developmental Stages

In the principal component analysis score plot, samples within the embryonic stage group exhibited a certain degree of separation trend. Verified by the three aforementioned quantitative analysis methods, the results demonstrated good intra-group sample reproducibility, with the observed separation falling within a reasonable range. Metabolomic analysis revealed distinct stage-specific metabolic characteristics during the early development of *M. volcano*, with the bile secretion pathway playing a core regulatory role across all three stages. In the comparison between the EMB and NAU, bile secretion, together with sulfur metabolism and pyrimidine metabolism, constituted the most significant differential pathways. In the comparison between EMB and CYP, however, bile secretion and mineral absorption emerged as the dominant enriched pathways. Some studies have shown that the secretion of bile-like substances in crustaceans can be adjusted accordingly to ensure the continuous supply of efficient energy substances such as lipids. These secreted substances can indirectly participate in humoral regulation; they help larvae maintain internal osmotic pressure stability and ensure that the developmental process is not disturbed by environmental stress [37,38,39,40]. This result suggests that bile acids not only participate in lipid digestion but may also regulate mineral absorption to supply key elements such as calcium and magnesium for carapace formation in the cyprid stage. This highly aligns with the morphological remodeling requirements of barnacles during the transition from nauplius to cyprid.

Further analysis of differential metabolite clustering showed that Cluster 3 metabolites were significantly enriched in pathways such as protein digestion and absorption, and ABC transporters. In contrast, Cluster 1 metabolites were concentrated in lipid synthesis-related pathways. This trend may reflect a shift in energy metabolism patterns: the embryonic stage relies on proteins provided by the mother as the main energy source; after entering the pelagic environment, the nauplius stage gradually shifts to lipid metabolism; and the cyprid stage then enhances lipid synthesis to store energy, preparing for the metabolic consumption during the subsequent permanent sessile process. Existing studies have confirmed that the esophagus and hindgut lumen of barnacle cyprids remain closed, leaving them incapable of feeding [41]. The metabolic costs incurred during their metamorphosis and permanent attachment are entirely dependent on energy reserves accumulated in earlier developmental stages. This finding is highly consistent with the significant enrichment of lipid synthesis pathways in the cyprid stage observed in the present study, and the results are also in line with the conclusions of Lou et al. regarding energy metabolism transitions in crustacean larvae [42].

Furthermore, metabolites in Cluster 2 were enriched in sphingolipid metabolism and apoptosis pathways, which might be directly associated with tissue remodeling during naupliar molting. In this study, sphingosine (VIP = 1.47, *p* = 0.002)—a core differential metabolite—was identified in this cluster. As a key intermediate product of sphingolipid metabolism, it not only provides structural support for cell membranes but also regulates cell proliferation or apoptosis through metabolic conversion. The specific accumulation of sphingosine in the naupliar stage is synchronized with the elimination of old tissues and differentiation of new tissues during molting. It may facilitate larval morphological remodeling by regulating cell fate [7,43,44].

### 4.2. Transcriptomic Networks Reveal Core Gene Modules for Developmental Regulation

A total of 9126 DEGs were identified through transcriptomic analysis, with their numbers gradually increasing along with the developmental progression. This indicates that the metamorphosis process of barnacles involves a sophisticated gene expression regulatory network. Among the four gene modules classified by WGCNA, the black module showed a significant correlation with CYP, and was significantly enriched in the Wnt signaling pathway as well as GO terms related to multicellular organism development.

As an evolutionarily conserved signaling pathway in crustaceans, the functions of the Wnt pathway are not limited to animal embryonic development and tissue polarity establishment; its high expression during the post-embryonic metamorphic stage, in the cyprid stage, is more likely to be involved in tissue and organ remodeling throughout barnacle metamorphosis. This finding is similar to the research results of Al-Aqeel et al: in the late larval development stage of *A. amphitrite*, the Wnt signaling pathway was significantly upregulated and proven to mediate key metamorphic processes [45]. In *A. amphitrite*, the Wnt signaling pathway is closely associated with signal transduction, chemotaxis, and sensory system development—functions that perfectly match the cyprid’s demand for body structural remodeling to achieve substrate attachment. Similar to the role of the Wnt pathway in regulating appendage differentiation and gland development in other barnacle species, the Wnt signaling pathway in cyprid larvae observed in this study may directly regulate antennule differentiation and the development of related organs.

The brown module was significantly correlated with NAU, with its functions concentrated in processes such as chitin metabolism and epidermal development. Chitin metabolism constitutes a core pathway in the early development of barnacle larvae. Existing studies have demonstrated that chitin serves not only as the primary structural component of the exoskeleton in insects but also in that of crustaceans, and its synthesis and degradation processes are directly involved in regulating larval molting and morphological remodeling [45,46,47,48,49]. The coordinated expression of 11 chitinase gene copies and chitin synthase genes within this module further confirms the central role of chitin metabolism in the multiple molting events of nauplius larvae, chitinases are responsible for degrading chitin in the old cuticle, while chitin synthases participate in the construction of the new cuticle. The dynamic balance between these two enzyme families ensures the smooth progression of larval body size enlargement and structural complexity. This regulatory pattern is consistent with research conclusions from closely related species. Transcriptomic and proteomic studies on *A. amphitrite* have shown that genes related to chitin-mediated epidermal development are significantly upregulated during nauplius larval development, which is closely associated with the periodic molting and cuticle renewal processes of planktonic larvae. In contrast, the expression levels of these genes decrease sharply when larvae transition to the cyprid stage [45]. Similarly, in *P. pollicipes*, chitin-binding function represents a significantly enriched GO term in the nauplius larval stage, and the expression levels of cuticle protein genes in nauplius larvae are also significantly upregulated [17]. In addition, the brown module was also enriched in lipid metabolism-related genes, forming a “transcriptome–metabolome” linkage with the activation of lipid synthesis pathways in the metabolome. This suggests that the coordinated regulation of gene expression and metabolic changes is key for nauplius larvae to adapt to pelagic life.

Both the green module and the yellow module were significantly correlated with EMB, and were enriched in functions such as cellular ion homeostasis and epithelial cell morphogenesis, respectively. The gene expression of these two modules gradually decreased with development, which may reflect the core physiological needs of the embryonic stage. Embryos rely on the maternal mantle cavity environment to maintain ion balance and construct basic body structures through epithelial cell morphology regulation. After entering the nauplius stage, these embryo-specific functions are replaced by the expression of pelagic adaptation-related genes, reflecting the stage-specific transition of gene functions during development. Building on the previous transcriptomic studies of *M. volcano*, this research further incorporated the embryonic stage into the scope of developmental analysis and revealed the modular characteristics of gene regulation via WGCNA. In contrast to the prior work of Yan et al., who identified settlement-related genes [28], and Bernot et al., who focused on clarifying the differences between larvae and adults [17], this study provides novel insights into the molecular mechanisms underlying the metamorphic development of barnacle larvae.

### 4.3. Co-Expression Network of Key Components for Mechanisms of Development Regulation

During the development of barnacle nauplius larvae, chitin plays an indispensable core role as a key material basis supporting their survival, growth, and stage transition [50]. As the core structural component of the nauplius exoskeleton, chitin forms a dense fibrous structure by binding to proteins. This structure not only provides physical protection for larvae—defending against mechanical damage and pathogen invasion, and maintaining the hemocoel shape to cope with osmotic pressure changes—but also supports the swing of appendages to ensure pelagic movement and feeding capabilities [50,51,52]. This study found that genes related to chitin metabolism are highly expressed during the nauplius stage. In the embryonic stage, chitin precursors accumulate to prepare for hatching; in the nauplius stage, both gene expression and precursor consumption increase to support multiple molts; and in the cyprid stage, the focus shifts to chitin synthesis to construct adhesion-related structures. This synergy between “gene regulation and material supply” ensures that chitin metabolism is functionally adapted to different developmental stages.

The larvae of most marine invertebrates must undergo a metamorphic process of transitioning from a planktonic larval stage to a benthic adult stage. Linoleic acid and its derivatives can promote larval shell calcification while providing energy for metamorphosis [53,54,55]. Studies have demonstrated that linoleic acid metabolism plays a critical role in the survival and growth of *Macrobrachium rosenbergii* larvae [56]. During the larval development of barnacles, metabolites related to linoleic acid and its derivatives detected by metabolomics within the linoleic acid metabolic pathway accounted for approximately 7.74% of the total number of fatty acid-related metabolites and around 0.7% of their relative content. Meanwhile, genes identified via transcriptomics—such as secretory phospholipase genes, CYP2J genes, and CYP3A genes—exhibit a correlation with the accumulation of linoleic acid derivatives including 9,10-dihydroxyoctadecenoic acid (9,10-DHOME) and 9,10-dihydroxy-12,13-epoxyoctadecanoic acid, as revealed by metabolomic analysis. These genes are responsible for the oxidative decomposition of linoleic acid and the synthesis of its derivatives, and their products may participate in larval metamorphosis by regulating apoptosis. For instance, the accumulation of 9(S)-HpODE in the cyprid stage may facilitate the degeneration of planktonic organs, thereby creating favorable conditions for the formation of a sessile morphology. This mechanism suggests that fatty acids such as linoleic acid act not only as energy sources but also potentially as signaling molecules involved in developmental regulation, which broadens the current understanding of the functions of fatty acids during barnacle metamorphosis.

The aforementioned pathways are closely associated with the core physiological processes of barnacle larval survival, development, and settlement, and thus can serve as potential targets for antifouling interventions. Wang et al. developed novel antifouling agents by targeting chitin synthase [57]. Similarly, inhibitors targeting the enzymes involved in the linoleic acid metabolic pathway can block the supply of energy and signaling molecules derived from linoleic acid, resulting in insufficient energy for larval metamorphosis and impaired organ remodeling, which may ultimately inhibit the settlement of barnacle larvae.

## 5. Conclusions

This study systematically clarified the molecular regulation and metabolic adaptation mechanisms during the early development of *M. volcano* using metabolomics and transcriptomics. A total of 3683 metabolites were identified, among which the bile secretion pathway runs through the entire early development process and gradually strengthens as larvae mature; it may support the “pelagic–sessile” transition by regulating lipid digestion and mineral absorption. At the transcriptomic level, 7234 DEGs were identified. The four gene modules arising from the WGCNA correspond to the core functions of different developmental stages. This study not only fills the research gap in the molecular mechanisms of barnacle larval development but also provides a theoretical basis for the prevention and control of marine fouling organisms. By targeting key pathways such as chitin metabolism and the linoleic acid metabolism pathway, new antifouling agents that specifically inhibit barnacle metamorphosis can be developed, reducing the damage by traditional chemical antifouling agents on the marine ecosystem. This has important scientific value and application prospects.

## Data Availability

The whole genome sequence data and metabolome data reported in this paper have been deposited in the Genome Warehouse in National Genomics Data Center, Beijing Institute of Genomics, Chinese Academy of Sciences/China National Center for Bioinformation, under accession number PRJCA028228 that is publicly accessible at https://ngdc.cncb.ac.cn/gwh (accessed on 25 January 2026).

## Supplementary Materials

The following supporting information can be downloaded at https://www.mdpi.com/article/10.3390/ani16030413/s1. Table S1. Primer sequence and product length of the target genes. Table S2. Metabolomics Analysis of Developmental Samples of *M.volcano*. Table S3. PERANOVA model fitting results. Table S4. Sequencing Quality Statistical Results. Figure S1. (A) OPLS-DA of Differential Metabolites Between Egg and Nauplii; (B) OPLS-DA of Differential Metabolites Between Egg and Cypris. Figure S2. (A) The number of differential accumulate metabolites (DAMs) in each cluster. The relative intensity of metabolites in cluster1 (B), cluster2 (C), and cluster3(D) during larval development of *M. volcano*. Figure S3. Top 20 KEGG pathway of cluster1 (A), cluster2 (B), and cluster3 (C). Figure S4. Module-phenotype association (The red-coloured cell indicated a positive correlation, and the blue-coloured cell means a negative correlation. The values in each cell indicated the correlation coefficient and *p*-value). Figure S5. Top 20 GO terms of cellular component in the WGCNA modules. Enriched GO of the black module (A), the brown module (B), the green module (C), and the yellow module (D). Figure S6. Top 20 GO terms of molecular function in the WGCNA modules. Enriched GO of the black module (A), the brown module (B), the green module (C), and the yellow module (D). Figure S7. Top 20 KEGG terms in the WGCNA modules. Enriched KEGG of the black module (A), the brown module (B), the green module (C), and the yellow module (D). Figure S8. KEGG Chitin Pathway Diagram. Figure S9. KEGG Linoleic Acid Pathway Diagram.
