# Supplementary material for: Comprehensive Analysis of Metabolome and Transcriptome Reveals Physiological Processes Related to Larval Development of Barnacles (Megabalanus volcano)
Source: Animals (Basel). 2026 Jan 28;16(3):413. doi: 10.3390/ani16030413 (PMC12897097; doi:10.3390/ani16030413)
Supplement: Supplementary file 1 [file animals-16-00413-s001.zip › animals-4035642-supplementary/Supplementary figures.pdf]

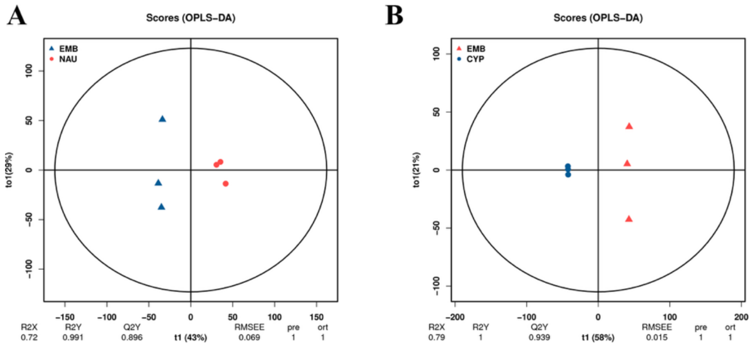

Figure S1. (A) OPLS-DA of Differential Metabolites Between Egg and Nauplii;(B) OPLS-DA of Differential Metabolites Between Egg and Cypris.

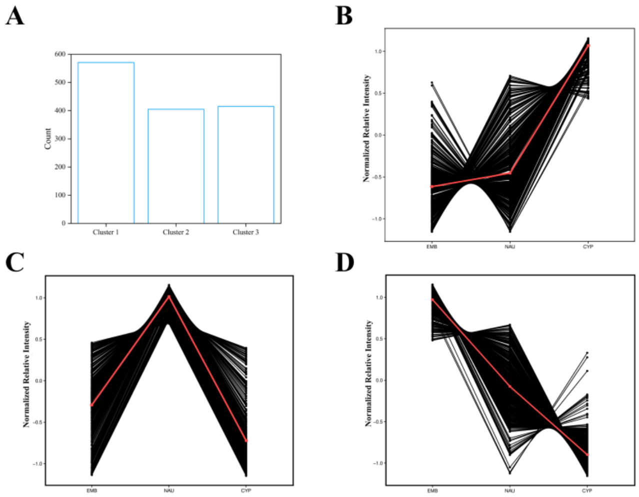

Figure S2. (A) The number of differential accumulate metabolites (DAMs) in each cluster. The relative intensity of metabolites in cluster1 (B), cluster2 (C), and cluster3 (D) during larval development of *M. volcano*.

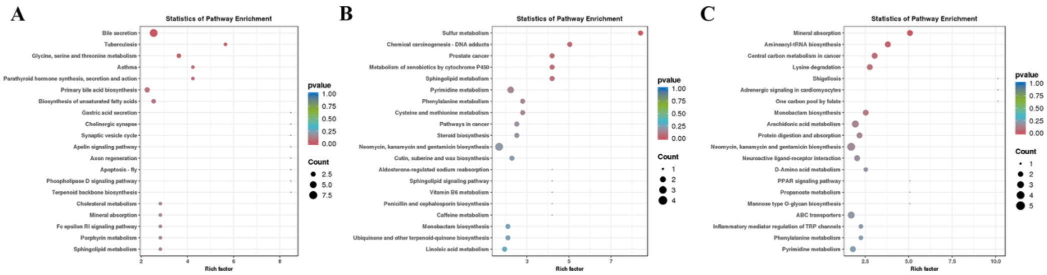

Figure S3. Top 20 KEGG pathway of cluster1 (A), cluster2 (B), and cluster3 (C).

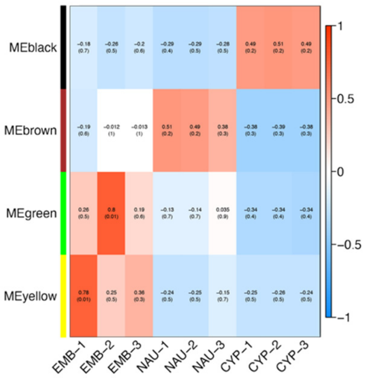

Figure S4. Module-phenotype association (The red-coloured cell indicated a positive correlation, and the blue-coloured cell means a negative correlation. The values in each cell indicated the correlation coefficient and p-value).

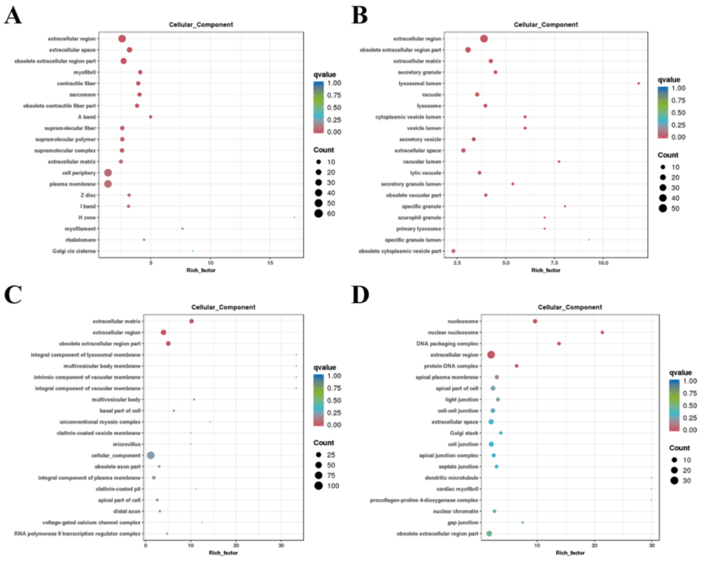

Figure S5. Top 20 GO terms of cellular component in the WGCNA modules. Enriched GO of the black module (A), the brown module (B), the green module (C), and the yellow module (D).

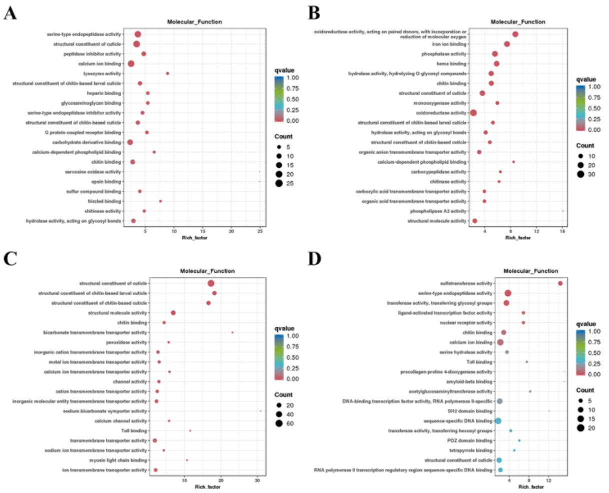

Figure S6. Top 20 GO terms of molecular function in the WGCNA modules. Enriched GO of the black module (A), the brown module (B), the green module (C), and the yellow module (D).

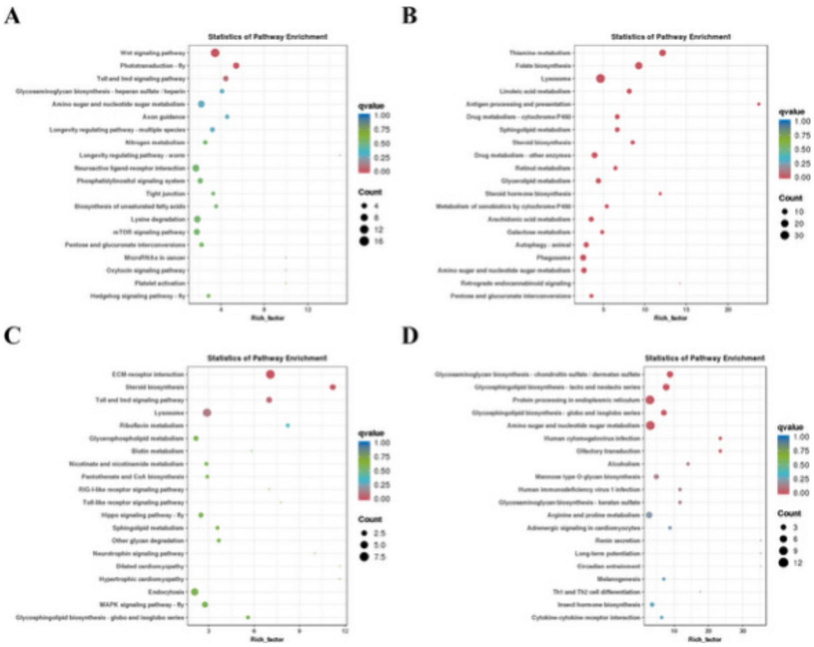

Figure S7. Top 20 KEGG terms in the WGCNA modules. Enriched KEGG of the black module (A), the brown module (B), the green module (C), and the yellow module (D).

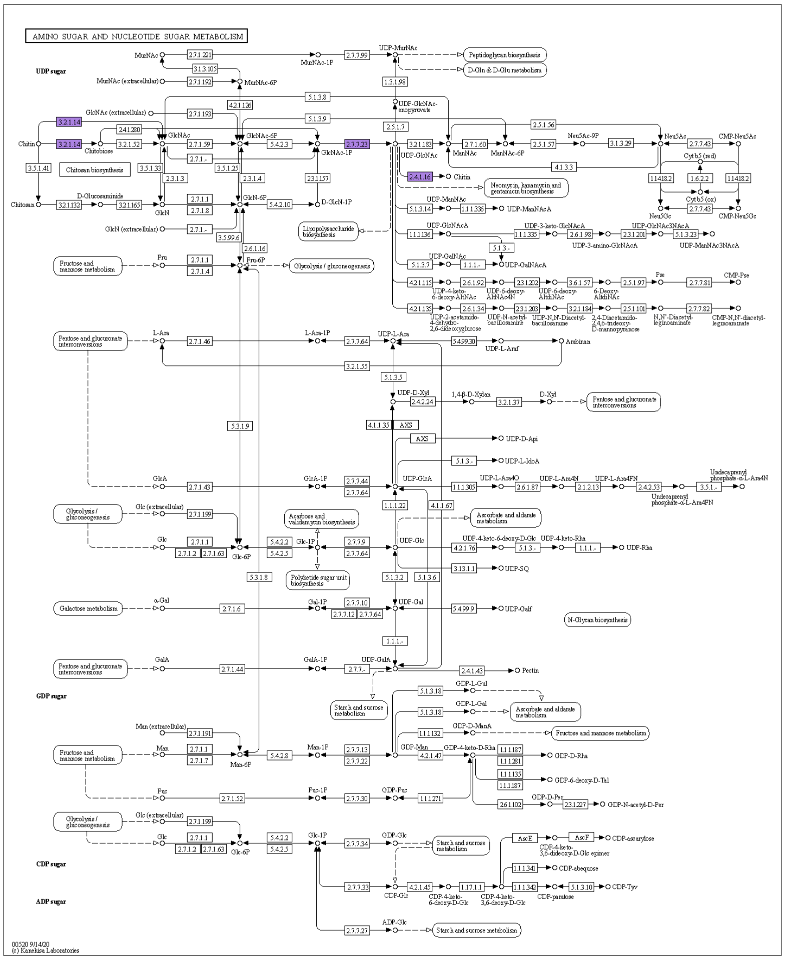

Figure S8. KEGG Chitin Pathway Diagram.

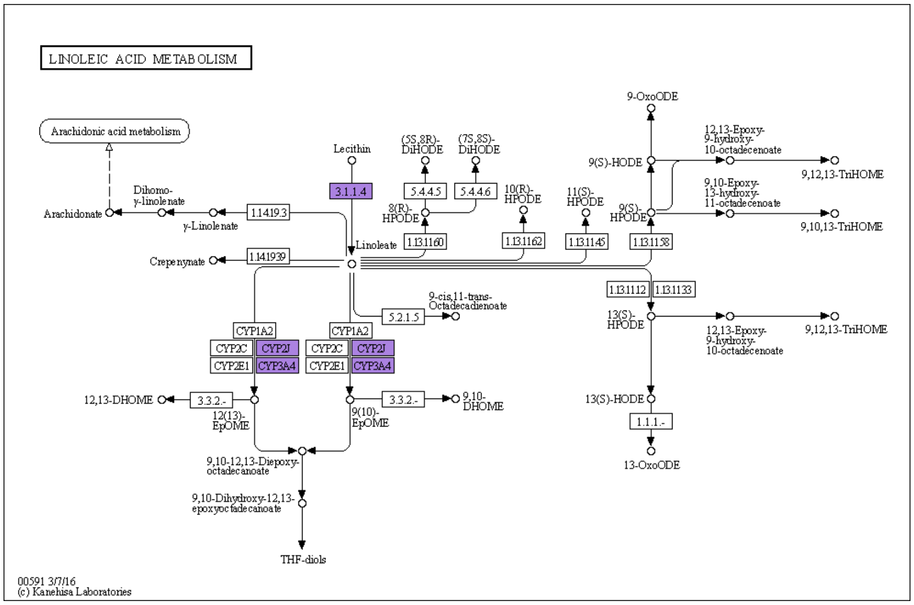

Figure S9. KEGG Linoleic Acid Pathway Diagram.
